# Strategies for Improvement of Lipid Production by Yeast *Trichosporon oleaginosus* from Lignocellulosic Biomass

**DOI:** 10.3390/jof7110934

**Published:** 2021-11-03

**Authors:** Marina Grubišić, Katarina Mihajlovski, Ana Marija Gruičić, Sunčica Beluhan, Božidar Šantek, Mirela Ivančić Šantek

**Affiliations:** 1 Faculty of Food Technology and Biotechnology, University of Zagreb, 10 000 Zagreb, Croatia; mgrubisic@pbf.hr (M.G.); amgruicic@pbf.hr (A.M.G.); sunbel@pbf.hr (S.B.); bsantek@pbf.hr (B.Š.); 2 Faculty of Technology and Metallurgy, University of Belgrade, 11 000 Belgrade, Serbia; kmihajlovski@tmf.bg.ac.rs

**Keywords:** oleaginous microorganism, corn cobs, biodiesel, cellulases, simultaneous saccharification and fermentation, separate hydrolysis and fermentation, enzyme recycle, *T. oleaginosus*

## Abstract

Microbial lipids have similar fatty acid composition to plant oils, and therefore, are considered as an alternative feedstock for biodiesel production. Oleaginous yeasts accumulate considerable amounts of lipids intracellularly during growth on low-cost renewable feedstocks such as lignocellulosic biomass. In this study, we cultivated yeast *Trichosporon oleaginosus* on hydrolysate of alkaline pretreated corn cobs. Different process configurations were evaluated and compared, including separate hydrolysis and fermentation (SHF) with cellulase recycle and simultaneous saccharification and fermentation (SSF) in batch and fed-batch mode. At low enzyme loading, the highest lipid concentration of 26.74 g L^−1^ was reached in fed-batch SSF fed with 2.5% (g g^−1^) substrate. Batch SHF was conducted for four rounds with recycling the cellulase adsorbed on unhydrolyzed lignocellulosic biomass. Thirty percent of cellulase saving was achieved for rounds 2–4 without compromising productivity and lipid yield. The addition of Tween 80 to lignocellulosic slurry improved the hydrolysis rate of structural carbohydrates in pretreated lignocellulosic biomass. Furthermore, supplementing the growth medium with Tween 80 improved lipid yield and productivity without affecting yeast growth. Oleaginous yeast *T. oleaginosus* is a promising strain for the sustainable and efficient production of lipids from renewable lignocellulosic feedstock.

## 1. Introduction

The latest biofuel production trends call for green and renewable raw materials at low prices and green technologies that can effectively replace today’s fossil fuel production. The European Union has set an ambitious target of 10% for the share of renewable energy (liquid biofuels, hydrogen, biomethane, green electricity) used in transport by 2020 and 14% in 2030, with advanced biofuels counting double to the target. In 2019, the average share of renewable energy in EU-27 used in transport was 8.9%. Among the member states, only Sweden, Finland, and the Netherlands (30.3%, 21.3%, and 12.5%, respectively) reached the set goal by 2019 [1,2]. Due to the COVID-19 crisis, the production of transport biofuels, biodiesel and bioethanol, has, for the first time, declined in the last two decades. In 2020, global production of biofuels reached 1677 thousand barrels of oil equivalent per day, while in 2019, 1790 thousand barrels of oil equivalent per day (b d^−1^) was produced [3]. The global production of biofuels is expected to recover to 2019 levels by the end of 2021 and reach 186.1 billion liters, or 3.21 million b d^−1^, in 2025 [4]. The dominant feedstock for biodiesel production is vegetable oils, with rapeseed oil as the most dominant one, followed by palm and soybean oils [4]. A set target of 10% renewable energy for transport by 2020 in the EU has driven up demand for cheap feedstocks, such as palm oil and soybean oils, mainly sourced from Asia and South America. Cultivating palm oil crops has led to deforestation, habitat loss and greater CO_2_ emissions than the fossil diesel it replaces [5]. Lipids produced by microorganisms are considered as a sustainable alternative to plant-derived lipids. Microbial lipids have several advantages over oil-seeds crops, including higher productivity, shorter generation time, and high oil content. Furthermore, the production is independent of climate conditions and arable land, less labour-intensive, and easy to scale up. Due to high lipid productivity, high lipid content in cell biomass, and rapid growth, oleaginous yeasts from the genera *Rhodosporidium, Cryptococcus, Lipomyces,* and *Rhodotorula*, are considered the most promising microorganisms for the industrial production of microbial lipids [6,7]. The most abundant lipid class in cell biomass, neutral lipids (primarily triacylglycerols), are stored in the form of intracellular lipid bodies at levels exceeding 70% of dry cell mass under favourable growth conditions. The most dominant fatty acids in yeast lipids are myristic (C14:0), palmitic (C16:0), palmitoleic (C16:1), stearic acid C18:0), oleic acid (C18:1), and linoleic acid (C18:2) [8,9]. Since the fatty acid composition of microbial lipids is similar to that of vegetable oils, they can be readily used for the production of biodiesel. Microbial biodiesel has similar physicochemical properties to petrol diesel and fulfils all requirements prescribed by standards for this type of fuel [10]. However, techno-economic analysis on biodiesel production showed that microbial lipids grown on typical industrial carbon sources such as glucose could not compete with vegetable oils. The carbon source used for yeast cultivation and oil production significantly contributes to a biodiesel price. The cost of lipid production using the yeast strain *Rhodosporidium toruloides* on glucose-based media was estimated to be USD 5.5 kg^−1^ of lipids, while biodiesel production cost was USD 5.9/kg. This study showed that glucose cost accounts for more than 35% of the total cost for biodiesel production [7]. Using the highly productive strain *R. toruloides* DEBB 5533 and sugar juice as substrate, Soccol et al. (2017) produced microbial biodiesel in the pilot-scale plant at a commercially viable price [11]. The estimated costs for biodiesel production from microbial lipids was lower than biodiesel from vegetable oils (USD 0.76 L^−1^ versus USD 0.81 L^−1^). However, most studies dealing with the heterotrophic microbial lipid production predict significantly higher prices of microbial lipids than vegetable oils (USD 1.72–5.9 kg^−1^ versus USD 0.5–1.9 L^−1^) [12]. Lipid titer is one of the critical factors affecting the viability of the process, and it can be improved by the increase of the cell density and lipid content using: specific cultivation strategies (continuous and fed-batch cultivation; [13]), optimizing substrate feeding, adjusting carbon to nitrogen ratio according to cell growth phase [13], engineering production strains [14] and dissolved oxygen adjustment [15]. Considerable cost savings could be accomplished by replacing costly substrates with the various renewable and inexpensive biomass and industrial by-products such as lignocellulosic biomass [16,17], sugar cane juice [11], molasses [18], biodiesel-derived glycerol [19], volatile fatty acids [20], whey [21], sewage sludge and wastewaters [19], waste cooking oil [20], etc. Plant-derived lignocellulosic biomass has been used as a source of energy-rich carbohydrates, which could be further efficiently converted to biofuels (ethanol, butanol) or feedstocks for their production (lipids for production of biodiesel) as well as bio-based chemicals [22]. In the production of bulk chemicals, high substrate concentrations are usually applied to improve the cost-effectiveness of the process and sustainability of the production. Running the process at high substrate loading has many advantages from an economic point of view; the efficiency of the bioprocess is increased, and the capital cost, labour, energy, and water demand are reduced [23]. Two types of cultivations are used in the lipid production of microbial lipids on lignocellulosic hydrolysate, separate hydrolysis and fermentation (SHF): simultaneous saccharification and fermentation (SSF). The main advantage of SSF over SHF is avoidance of cellulase inhibition by end-product and decrease of contamination risk by conducting the cultivation and enzyme hydrolysis in a single bioreactor. However, key process performance variables in SSF, including lipid concentration, lipid yield and productivity, are usually lower than that seen in fed-batch SHF. The maximal lipid yield of 159 mg g^−1^ of pretreated corn stover was obtained using yeast *Cryptococcus curvatus*. by Gong et al. (2014). Lipid concentration and productivity in this batch SSF was 16 g L^−1^ and 4.7 g L^−1^ d^−1^, respectively [24]. Similarly, Ivancic Santek et al. performed SHF and SSF cultivations with yeast *T. oleaginosus* DSM 11815 using corn cobs as a carbon source [17]. Maximal lipid productivity and concentrations of 2.4 g L^−1^ d^−1^ and 13.45 g L^−1^ d^−1^, respectively, was obtained at considerably smaller inoculum size compared to the previous study. *T. oleaginosus* efficiently accumulates lipids in the glucose-based medium. Lipid content in cells grown on a glucose-based medium under nitrogen deprivation usually exceeds 50% of dry cell weight, while lipid productivity reaches maximal 0.67 g L^−1^ h^−1^. Due to the favourable fatty acid profile, *T. oleaginosus* is considered as a promising strain for the production of microbial lipids and biodiesel [8].

Running the SSF cultivation at lower initial substrate loading with gradual additions of the lignocellulosic substrate could enhance the lipid yield and decrease the enzyme loading. A similar strategy is successfully applied in bioethanol production from lignocellulosic biomass, resulting in high product yield and high cumulative substrate loading [23,24]. However, productivities and lipid titres in yeast cultures grown on lignocellulosic hydrolysates are lower than those obtained in synthetic media with glucose as carbon sources, especially at high substrate loadings due to increased inhibitors concentration. Efficient conversion of lignocellulosic biomass to fermentable sugars includes a pretreatment process that improves the susceptibility of cellulose to the cellulolytic enzyme. Several lignocellulose-derived inhibitors are formed during pretreatment, including furan aldehydes, aliphatic carboxylic acids and phenolic compounds that affect cell growth, lipid accumulation and cellulose hydrolysis. The concentration and nature of the inhibitors generated during pretreatment depend on the type of pretreatment method, conditions and type of feedstock [25,26].

In this study, we applied different strategies for improving the lipid productivity of *T. oleaginosus* grown on lignocellulosic biomass, including cultivation method (batch and fed-batch), the process configuration (SHF and SSF), the addition of surfactant, and enzyme recycling. The hydrolysis of lignocellulosic biomass in SSF was conducted at low cellulase loading for prolonged reaction times to obtain higher yields and product concentrations. A decrease of freshly added enzyme in SHF was obtained by recycling the cellulases bound to the unhydrolyzed biomass in subsequent hydrolysis steps.

## 2. Materials and Methods

### 2.1. Materials

Corn cobs are agro-wastes grown in Zagorje county, Croatia and harvested in October 2016. Dried lignocellulosic biomass was shredded to smaller pieces using a garden shredder (Hurricane HMH 200, Germany), cut with a cutting mill (Retsch SM 2000, Germany) and sieved through a 1 mm screen. Biomass was stored in the plastic container at room temperature (18–21 °C) in the dark. It contained approximately 5% (g g^−1^) of moisture.

Commercially available enzyme mixes Celluclast 1.5 L (Sigma, St. Louis, MO, USA), Viscozyme L (Sigma, St. Louis, MO, USA), and Cellic CTec2 (Novozymes, Copenhagen, Denmark) were used for hydrolysis of lignocellulosic biomass. The cellulase activity was determined according to the International Union of Pure and Applied Chemistry [27]. The filter paper activity of Celluclast 1.5 L and Viscozyme L was 65.2 and 22.2 filter paper units mL^−1^ (FPU mL^−1^), respectively. Glucose was purchased from Carl Roth GmbH (Karlsruhe, Germany), yeast extract and peptone were purchased from Merck Millipore (Darmstadt, Germany), mineral salts were obtained from Kemika (Zagreb, Croatia), and chloroform and methanol were purchased from Fisher Scientific (Cleveland, OH, USA).

### 2.2. Microorganism

In the present study, yeast *T. oleaginosus* DSM 11815 was used for lipid production. Yeast was grown on YPD agar slants (10 g L^−1^ yeast extract, 20 g L^−1^ glucose, 20 g L^−1^ peptone and 20 g L^−1^ agar) at 30 °C. The inoculum was cultivated in liquid YPD medium at 30 °C for 48 h on a rotary shaker at 180 min^−1^.

### 2.3. Pretreatment

Lignocellulosic biomass was subjected to alkali-pretreatment before cellulose hydrolysis. Ground corn cobs were mixed with 3% sodium hydroxide in a solid to liquid ratio of 1:8 (g of the dry weight of untreated lignocellulosic biomass per mL of alkali solution) and autoclaved at 121 °C for 30 min. After separation by filtration, the filter cake was washed with demineralized water to pH = 7.0–7.5. The pretreated lignocellulosic biomass was stored at −20 °C.

### 2.4. Effect of Substrate Loading on Lipid Yield 

The effect of substrate loading on the productivity of the simultaneous saccharification and fermentation (SSF) process with a prehydrolysis step was studied. Batch cultivations were performed in 500 mL Erlenmeyer flasks at 5%, 7.5%, 10%, 12.5%, 15% and 20% (g g^−1^) substrate loading. Growth media contained phosphate buffer (50 mM, pH = 5.0), trace elements and nutrient solution. Macro and micronutrients were added to obtain the final concentrations: (trace elements solution) 40 mg L^−1^ CaCl_2_·2H_2_O, 5.5 mg L^−1^ FeSO_4_·7H_2_O, 0.52 mg L^−1^ citric acid monohydrate, 1 mg L^−1^ ZnSO_4_·7H_2_O, 0.76 mg L^−1^ MnSO_4_·H_2_O and 200 μL 9 M H_2_SO_4_. and (nutrient solution) 1.0 g L^−1^ yeast extract 0.95 g L^−1^ Na_2_HPO_4_, 2.7 g L^−1^ H_2_PO_4_, 0.2 g L^−1^ MgSO_4_·7H_2_O and 0.1 g L^−1^ EDTA [8]. Optimal initial carbon to nitrogen ratio of 207.4 mol mol^−1^ was obtained by supplementing growth media with the appropriate amount of NH_4_Cl. The mixture of Celluclast 1.5 L and Viscozme L was added to start prehydrolysis. The total cellulase loading was 5 FPU per gram of glucan. Based on the previous experiments, Viscozme L was added at 0.1 mL per gram of dry weight of the substrate, while the volume of Celluclast 1.5 L was calculated by substracting FPU of Viscozyme loading from the total cellulase loading [17]. After 12 h of prehydrolysis at 40 °C and gentle mixing on a rotary shaker (50 rpm), the lignocellulosic slurry was cooled down to room temperature and inoculated with 10% (*v v*^−1^) of *T. oleaginosus* culture. The cultivations for each substrate loading were performed in duplicates. Cultures were incubated at 30 °C on an orbital shaker at 180 min^−1^. The cultivation time and time-points for the sampling were estimated based on growth rates and substrate consumption rates of *T. oleaginosus* on glucose and xylose. Change of culture densities and homogeneities were observed visually. The solid residue containing unhydrolyzed corn cobs and yeast biomass was separated by centrifugation. The fermented broth was centrifuged at 8000× *g* for 30 min. The supernatants were further analyzed for monosaccharides content, while the solids were freeze-dried. The concentration of total solids during cultivation was calculated based on the dry weight of solid residue and the culture broth volume. Lipid content in the solid residues was determined gravimetrically after the extraction of total lipids. The concentration of lipids in the cultures was calculated based on lipid content and concentration of solids. 

### 2.5. Fed-Batch SSF at Low Enzyme Loading

The effect of total substrate loading and amount of substrate feed on process efficiency of the fed-batch SSF was investigated. The cultivations were conducted using two feeding strategies. Initial substrate loading was 5% (g g^−1^) in both cultivations. In the first cultivation (FB_1), culture was fed with 5.0% (g g^−1^) substrate, and cumulative substrate loading at the end of the process was 15% (g g^−1^). In the second cultivation (FB_2) culture was fed with 2.5% (g g^−1^) substrate, while cumulative substrate loading was 20% (g g^−1^). The enzyme hydrolysis and lipid production experiments were performed in 500 mL Erlenmeyer flasks containing 100 g cultivation media. Culture media contained 50 mM phosphate buffer (pH = 5.0), solution of trace elements and nutrient solution as described for batch SSF experiment. Enzyme loading was 5 FPU per gram of glucan of Celluclast 1.5 L and Viscozyme L (0.1 mL g^−1^ of substrate dry weight). A total amount of enzyme preparations was calculated based on the cumulative substrate loading (15% and 20%, g g^−1^) and added at the beginning of the process. The prehydrolysis, inoculation and cultivation were performed as described in the previous experiment.

### 2.6. Fed-Batch SSF at High Enzyme Loading in the Presence of Tween 80

The next two fed-batch SSF experiments were performed at a high enzyme loading of 30 FPU Cellic CTec2 per gram of glucan at two molar carbon to nitrogen ratios (C:N, mol mol^−1^) of 207.4 (FB_3) and 38.7 mol mol^−1^ (FB_4) [8]. Initial substrate loading in both cultivations was 7.5% (g g^−1^). After 12 h of prehydrolysis, the lignocellulosic hydrolysate was inoculated with 10% (*v v*^−1^) yeast culture. Cultures were fed with 2.5% (g g^−1^) substrate on day 0, 6, 9, 12 and 15. Five substrate additions were equivalent to cumulative total substrate loading of 20% (g g^−1^). The total amount of cellulolytic enzyme for lignocellulose hydrolysis was calculated based on cumulative substrate loading in the process, and it was added at the beginning of the process. The prehydrolysis step, inoculation and cultivation of microorganisms were performed under the same conditions used in the previous fed-batch SSF experiments. The composition of culture media was also the same as in the previous fed-batch experiment conducted at low enzyme loading (Section 2.5) except for the C: N ratio in FB_4 fed-batch cultivation. Culture FB_4 was additionally supplemented with 1.5 g L^−1^ of ammonium chloride. Cultivation media also contained 6.5 g L^−1^ Tween 80. 

### 2.7. Effect of Tween 80 on Enzyme Hydrolysis and Lipid Production 

Enzymatic digestibility of pretreated corn cobs was performed in 50 mM citrate buffer (pH = 4.8) containing 1% of glucan per gram of slurry (g g^−1^), 25 FPU of Celluclast 1.5 L g^−1^ glucan and 100 mg L^−1^ ampicillin according to Ivancic Santek et al. [17]. The lignocellulosic slurries were supplemented with 0.5–25 g L^−1^ Tween 80. The mixtures were gently mixed using a magnetic stirrer for three days at 40 °C. The samples were withdrawn at 0, 3, 6, 24, 48 and 72 h. Withdrawn samples were immediately heated for 10 min in a boiling water bath, cooled and centrifuged at 10 000 rpm for 10 min. Samples were filtered through a 0.22 μm syringe filter (nitrocellulose, Sartorius, Germany) and analyzed for glucose and xylose content by HPLC. 

The set of batch cultivations of *T. oleaginosus* were carried out to investigate the effect of Tween 80 on cell growth and lipid production. Cultivations were performed in 500 mL Erlenmeyer flasks with 100 mL growth media containing 50 g L^−1^ glucose as a carbon source. Growth media contained 50 mM citrate buffer (pH = 5.0), solutions of trace elements and nutrient solution (see Section 2.4). Initial C:N ratio was 207.4 mol mol^−1^. After inoculation with 10% (*v v*^−1^) of *T. oleaginosus* preculture, cultures were incubated at 30 °C on an orbital shaker at 180 min^−1^. After cell biomass separation by centrifugation, culture supernatants were analyzed for glucose. Yeast biomass was washed with water and freeze-dried. The lipid content in cell biomass was determined using a modified Schneiter and Daum extraction protocol [8,28,29].

### 2.8. Separate Hydrolysis and Fermentation (SHF) with Cellulase Recycling

**Figure 1:**
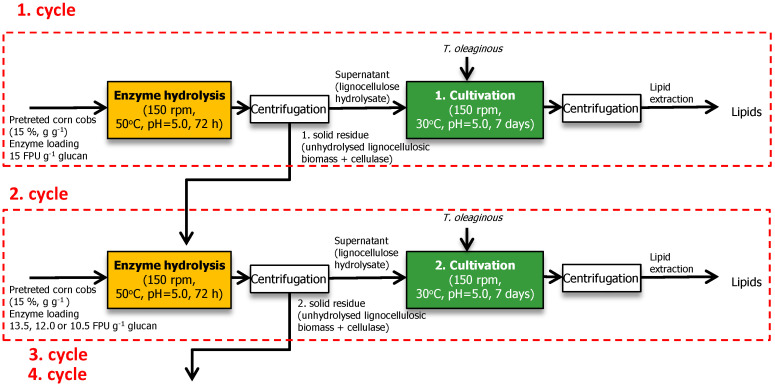
Flow diagram for batch SHF with enzyme recycle.

Separate hydrolysis and fermentation were performed in duplicate. Enzymatic hydrolysis of pretreated corn cobs was performed in 50 mM citrate buffer (pH = 5.0) containing 15% (g g^−1^) of the pretreated substrate. Enzyme loading used for the first cycle was 15 FPU of Cellic CTec2 g^−1^ glucan, while cycles 2, 3 and 4 were conducted at 13.5, 12.0 and 10.5 FPU g^−1^ glucan (Figure 1). The enzyme was previously sterilized by filtration using membrane filtration (0.2 μm, Sartorius). Reactions were conducted in 300 mL Erlenmeyer flasks for 72 h at 50 °C followed by centrifugation. The unhydrolyzed solids were separated by centrifugation and added to the next enzymatic reaction mixture, increasing the volume from cycle to cycle. The supernatant of hydrolysate was supplemented with trace elements and nutrient solution as described for batch experiment and inoculated with *T. oleaginosus* grown of YPD medium. Cultivation was conducted at 30 °C and 180 min^−1^ on an orbital shaker for a week. 

### 2.9. Chemical Analysis of Lignocellulosic Biomass

The pretreated biomass was subjected to chemical compositional analyses. Carbohydrate content, acid-insoluble lignin (AIL) and acid-soluble lignin (ASL) were determined following the National Renewable Energy Laboratory Chemical Analysis and Testing Standard Procedure [30]. First, lignocellulosic biomass was dried to constant weight at 105 °C and ground to a fine powder. After two-stage sulfuric acid hydrolysis, lignocellulosic hydrolyzates were analyzed for acid-soluble lignin (ASL) by spectrophotometric analysis and sugar monomers by HPLC. The insoluble residue that remained after the hydrolysis was reported as acid-insoluble lignin (AIL). 

### 2.10. High-Pressure Liquid Chromatography (HPLC) Analysis

The concentration of released sugars (glucose, cellobiose, xylose and arabinose) was determined by HPLC (Shimadzu, Kyoto, Japan) using a Supelcogel C610H column (300 mm × 7.8 mm; Supelco Analytical, Bellefonte, PA, USA) with matching guard column at 55 °C and 0.1% phosphoric acid as eluent at a flow rate of 0.5 mL min^−1^. The sugars were detected by a refractive index detector (Shimadzu RID-10).

### 2.11. Determination of Lipid Content

The lipid content in freeze-dried cell biomass and the solid residue was determined by the modified Schneiter and Daum method [8,28]. In a Pyrex glass tube, 200 mg of sample was resuspended in a mixture of solvents (0.3 mL water, 2 mL methanol and 4 mL chloroform). After an overnight extraction at 30 °C, undissolved solids were removed by filtration through sintered glass funnels. The remaining solids were washed twice with a mixture of solvents previously used for lipid extraction. Extracts were further purified using 0.0034% *w w*^−1^ MgCl_2_ (20% of the extract volume, *v v*^−1^). The chloroform phase was transferred into a new Pyrex glass using a syringe and needle. The organic solvent was evaporated under a nitrogen flow and dried to constant weight at 105 °C. Lipid content in cell biomass or recovered solids was calculated as a percentage of the dry weight.

## 3. Results and Discussion

### 3.1. Chemical Composition of Pretreated Biomass

The most abundant structural polymers in corn cobs are glucan, xylan, and lignin (Table 1). Lignin and hemicellulose form a physical barrier that prevents cellulases from accessing their substrate, significantly reducing the hydrolysis efficiency of cellulose. Therefore, the lignocellulosic biomass is subjected to a pretreatment process before the enzymatic digestion. The pretreatment process improves the monosaccharide yield and hydrolysis rate of carbohydrates but also increases production costs. Besides acid pretreatment, alkali-pretreatment is commonly used to enhance cellulose digestibility of lignocellulosic biomass with low lignin content, such as herbaceous crops and agricultural residues (wheat straw). This type of pretreatment is moderately effective for hardwood and completely ineffective for softwoods with high lignin content [31]. Pretreatment makes the carbohydrates more accessible to cellulase and hemicellulases by breaking the chemical bonds between lignin and carbohydrates and disrupting the lignin structure. Alkaline hydrolysis of intermolecular ester bonds cross-linking xylan hemicellulose and other lignocellulosic components (e.g., lignin, other molecules hemicellulose) decreases the hemicellulose and lignin content and makes cellulose microfibrils more accessible to cellulases. Alkali-pretreatment effectively solubilizes lignin living more unhydrolyzed hemicellulose in lignocellulosic biomass than acid pretreatment. It also changes the physical properties of lignocellulosic biomass, including surface area, porosity, and crystallinity [32].

In our study, alkali-pretreatment effectively reduced acid-soluble and acid-insoluble lignin in corn cobs by 85% and 75%, respectively (Table 1). The increase of glucan content (4.5%) in pretreated biomass accompanied the minor loss of hemicellulose (3.6%), which *T. oleaginosus* could also use as a carbon source for the growth and lipid synthesis after hydrolysis to simple sugars. The chemical composition of untreated and pretreated corn cobs was comparable to literature data [17,33]. Jiang et al. (2019) treated corn cobs with sodium hydroxide (0–3%, g g^−1^) at 80 °C for 1 h. Pretreated biomass decreased the lignin content from 5.1–87.6%, depending on the weight percentage of sodium hydroxide. Treatment with 3% alkali decreased the lignin content by 87.6% (g g^−1^) and increased cellulose to content from 34.4 (raw corn cobs) to 69.4% (pretreated corn cobs). Hemicellulose content moderately decreased proportionally to alkali weight percentage [33].

### 3.2. Effect of Substrate Loading on Lipid Yield

**Figure 2:**
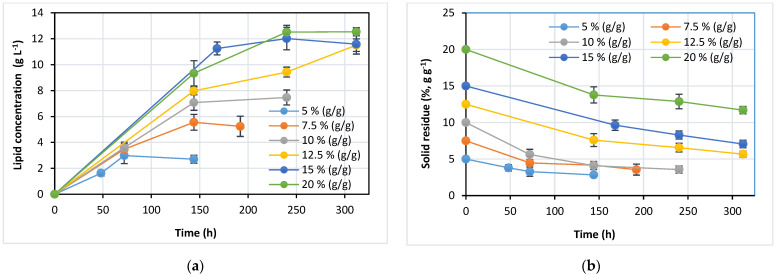
Effect of substrate loading on lipid yield in SHF batch cultivation: (**a**) lipid concentration profiles during cultivation; (**b**) solid residue (unhydrolyzed lignocellulosic biomass + yeast biomass) profiles in culture broth during cultivation.

In order to investigate the effect of substrate loading on product yield, batch SSFs were performed at 5 to 20% (g g^−1^) of corn cobs (Figure 2). The free liquid in the culture broth was significantly reduced at higher solid content (above 15%, g g^−1^ of substrate loading), especially at the prehydrolysis step. Swollen corn cobs grains retained their structure for 1–2 days, with polysaccharide fibers merely taking on liquid. Due to enzyme hydrolysis, the consistency of the culture broth gradually changed to a viscous liquid free of grain particles. A significant change in rheology was observed in culture broths at solid loadings over 15% (g g^−1^). During the first phase of cultivation, the appearance of lignocellulosic slurries changed from the solidified substrate to clumps with unstirred areas. Polysaccharides in lignocellulosic biomass adsorb water molecules by forming hydrogen bonds with hydroxide groups on the sugar rings. Cellulose fibers bind a free liquid from growth media, causing the fiber swelling and reducing available free liquid. The water-holding capacity of cellulose fibers can be from 3 up to 7 g of water g^−1^ of dry weight depending on fiber length and particle distribution [34]. Similar to cellulose, hemicellulose in lignocellulosic biomass also has a high water-holding capacity. Both polysaccharides influence the rheology of the lignocellulosic slurries and determine their mixing behaviour, mass transfer of enzymes and substrates and reaction efficiency [35].

A positive effect of substrate loading on lipid yield was observed below 12.5% (g g^−1^) (Figure 2a). An increase of substrate loading above 12.5% (g g^−1^) only moderately increased the final lipid concentration. The maximal product concentration of 12.52 g L^−1^ was obtained at the highest substrate loading of 20% (g g^−1^). During the first day of cultivation, the concentration of glucose and xylose drastically decreased due to intensive cell growth and product synthesis (results not shown). After the second day of cultivation, glucose and xylose concentrations remained below 1.0 and 1.5 g L^−1^, respectively, at all substrate loadings. The low concentration of monosaccharides in the phase of intensive lipid accumulation indicates rapid consumption of sugars by yeast cells, excluding the possibility of cellulases inhibition by end-products. Nevertheless, low sugar concentration shows that microorganism growth and lipid production were limited by carbon source, affecting the overall lignocellulose-to-lipid conversion efficiency.

The concentration of suspended solids in the culture broth was also determined during the cultivation. They contained yeast biomass in addition to unhydrolyzed lignocellulosic biomass (Figure 2b). The suspended solids profile shows two distinct phases. The first phase is characterized by a faster decrease of solid residue due to hydrolysis of substrate by cellulases and correlates well with faster lipid synthesis (Figure 2a). In the second phase, a slow drop of solid residue concentration was accompanied by slower lipid synthesis, probably due to the low carbon source concentration.

Bioprocess efficiency parameters are presented in Table 2. The increase of substrate loading from 5% to 12.5% (g g^−1^) enhanced the lipid yield and recovery. However, substrate loading exceeding 12.5% (g g^−1^) decreased both efficiency criteria. The maximal lipid yield of 92.1 mg g^−1^ (corresponds to 32.72% of theoretical lipid yield) was obtained with substrate loading of 12.5% (g g^−1^). Increased substrate loading is a common strategy applied in producing lignocellulosic biofuels to improve the product yield. A similar effect of high-substrate loading on enzyme hydrolysis of pretreated rapeseed straw and product accumulation has been observed in bioethanol production [36]. An increase of substrate loading up to 20% (g g^−1^) positively affected product end concentration. However, the maximal bioethanol yield (g of product g^−1^ of the substrate) and bioethanol productivity was obtained at substrate loading of 15% (g g^−1^). Dai et al. (2019) studied the effect of substrate loading (10–20%, g g^−1^) on lipid production using yeast *Rhodosporidium toruloides* in the SSF process with prehydrolysis step [37]. Similar to our results, an increase of acid pretreated corn stover loading up to 12.5% (g g^−1^) improved the lipid yield (0.8 g g^−1^) and lipid concentration (10.1 g L^−1^). However, substrate loadings above 12.5% (g g^−1^) moderately enhanced the lipid concentration and decreased the lipid yield. Maximal lipid concentration of 12.5 g L^−1^ was obtained at substrate loading of 20.0% (g g^−1^), while lipid yield decreased to 0.62% (g g^−1^). Similarly, Liu et al. (2012) observed a 30% increase of lipid concentration in SSF with yeast *Trichosporon cutaneum* when substrate loading (pretreated corn stover) was increased from 10 to 15% (g g^−1^) [38].

### 3.3. Fed-Batch SSF at a Low Enzyme Loading

The fed-batch strategy in SSF enables overcoming the issues with irregular mixing, low mass transfer of enzyme and substrate, and low product yield characteristic for batch cultivation at high-substrate loading [35,39]. Fed-batch cultivations were conducted at 15% and 20% substrate loading using two feeding strategies to improve the lipid yield. Low initial substrate loading (5%, g g^−1^) and sequential addition of substrate at 2.5% and 5% (g g^−1^) during cultivation enabled to overcome problems with high viscosity of culture media previously observed in batch SSF and enabled more efficient mixing and mass transfer. Two substrate additions at 5% (g g^−1^; FB_1 culture) and six additions at 2.5% (g g^−1^; culture FB_2) resulted in accumulative substrate loading of 15% and 20% (g g^−1^), respectively. The total enzyme amount (5 FPU *g*^−1^ glucan) calculated on cumulative substrate loading was supplied at the beginning of the prehydrolysis step. After a short prehydrolysis step, the lignocellulosic slurry was inoculated with yeast. A similar strategy of feeding and early addition of a total amount of cellulases (based on cumulative substrate loading) was successfully applied in the production of bioethanol from waste paper, increasing the yield of ethanol (11.6%, *v v*^−1^) and cumulative substrate loading (65%, g g^−1^) [40]. The time for substrate additions depended on the viscosity of the culture broth, which was estimated by visual monitoring. Sequential addition of substrate prevented from forming substrate clumps and slurry inhomogeneity usually characteristic for high gravity fermentation. As a result, a high enzyme to substrate ratio at the beginning of the fed-batch SSF enabled a faster decrease of viscosity than the batch SSF cultivations previously conducted. After ten days of cultivation, the cumulative substrate loading was 15% (g g^−1^) in both culture broths. However, the lipid concentration and productivity in FB_2 culture were slightly higher than in FB_1, suggesting that the feeding regime with 2.5% (g g^−1^) substrate was more favourable for lipid accumulation (Table 3). Two more substrate additions at 2.5% (g g^−1^) in FB_2 culture rise cumulative substrate loading to 20% (g g^−1^). The final lipid concentration of 27.18 g L^−1^ is the highest reported value obtained in the SSF process. During growth, glucose and xylose concentrations were below 1.5 and 3 g L^−1^, respectively, allowing substrate inhibition free enzymatic hydrolysis. However, low concentrations of fermentable sugars suggest that growth and lipid production was limited by carbon source. In accordance with the observed, moderate values of lipid production were obtained during cultivation (Table 3).

Gong et al. (2014) performed batch SSF with yeast *C. curvatus* using alkali-pretreated corn stover at 10% (g g^−1^) substrate loading and obtained 15.9 g L^−1^ lipids. Higher productivity (Pr= 4.69 g L^−1^ d^−1^) obtained in this study was probably due to higher enzyme loading (10 FPU g^−1^ of pretreated biomass) and higher inoculum concentration (approximately nine times higher, 7.2 g L^−1^) [24]. Ivancic Santek et al. obtained 13.5 g L^−1^ lipids using *T. oleagnosus* in fed-batch SSF with alkali-pretreated corn cobs as substrate. High enzyme loading (30 FPU g^−1^ glucan) enabled efficient hydrolysis of structural carbohydrates and a high concentration of fermentable sugars, which supported fast growth and lipid production. Lipid productivity was moderately higher (2.43 g L^−1^ d^−1^) [17]. Obtained results show that integrating the fed-batch enzymatic hydrolysis and microorganism cultivation leads to higher lipid titers at relatively low enzyme loading. The carbon source concentration can be increased by improving the enzyme hydrolysis rate through increasing enzyme loading, which further positively affects product synthesis and enhances the productivity of the process [17].

### 3.4. Effect of Tween 80 on Enzyme Hydrolysis and Lipid Production

**Figure 3:**
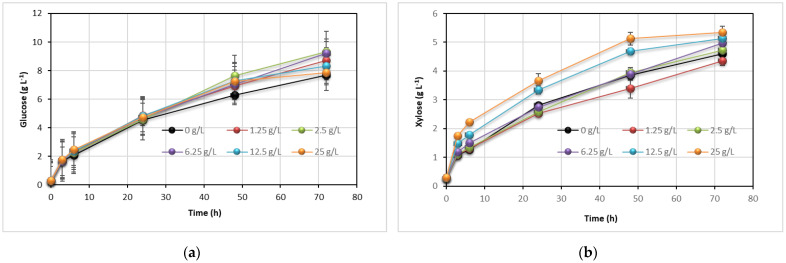
Effect of Tween 80 on the enzymatic hydrolysis of glucan (**a**) and xylan (**b**) in pretreated corn cobs. Reaction conditions: 1% (g g^−1^, g glucan per g of lignocellulosic slurry) substrate loading in citrate buffer pH=5.0, 25 FPU of Celluclast 1.5 L g^−1^ glucan and 100 mg L^−1^ of ampicillin.

Next, we studied the effect of a non-ionic surfactant on the hydrolysis rate of carbohydrates in pretreated corn cobs. Kinetics of glucan and xylan hydrolysis in the presence of Tween 80 over a concentration range from 0 to 25 g L^−1^ was determined. Glucose and xylose concentrations in reaction mixtures are presented in Figure 3. The Tween 80 enhanced the hydrolysis rate for both structural carbohydrates and improved both sugar yields. During the first 24 h, xylose concentration increased more rapidly compared to that of glucose. At the same time, negligible differences in glucose concentrations were observed at different Tween 80 concentrations. The highest glucose concentration of 9.31 and 9.22 g L^−1^ was observed in the presence of 2.5 and 6.25 g L^−1^ of the surfactant, respectively, after 72 h. Concentrations of Tween 80 above 6.25 g L^−1^ slightly decreased glucose yield. Similar observations were reported for common reed at 2% and 4% of Tween 80 [41]. On the contrary, xylose yield increased proportionally with the surfactant concentration. The highest xylose concentration of 5.34 g L^−1^ was obtained at 25 g L^−1^ of Tween 80 after 72 h. Low monosaccharides yields were probably due to inefficient xylanase activity of enzyme preparation and the presence of remaining lignin [26,42].

Most of the studies reported in the literature have been performed at low substrate loading or high enzyme loading that support the high substrate conversions rates. However, this strategy increases the costs and feasibility of lipid production, and therefore, it is not applied on an industrial scale. The addition of Tween 80 improved glucose and xylose yield (21% and 16%, respectively) and increased the hydrolysis rate. Considering the low price of Tween 80, supplementing an enzyme reaction with this surfactant could also raise the sugar yield and improve economic performance [42].

Non-ionic surfactants, Tween 80, is widely used as a component of microorganism growth media. It improves the growth of microorganisms [43,44,45] and the synthesis of different products such as pigments [46], extracellular enzymes [47], ethanol [48,49] and lipids [43,46]. Although the mechanism of its action in the cell is still not fully elucidated, conducted studies suggest that Tween 80 improves the membrane permeability [47]. Tween 80 affects the cell membrane composition, physical properties and its function [43,44].

**Figure 4:**
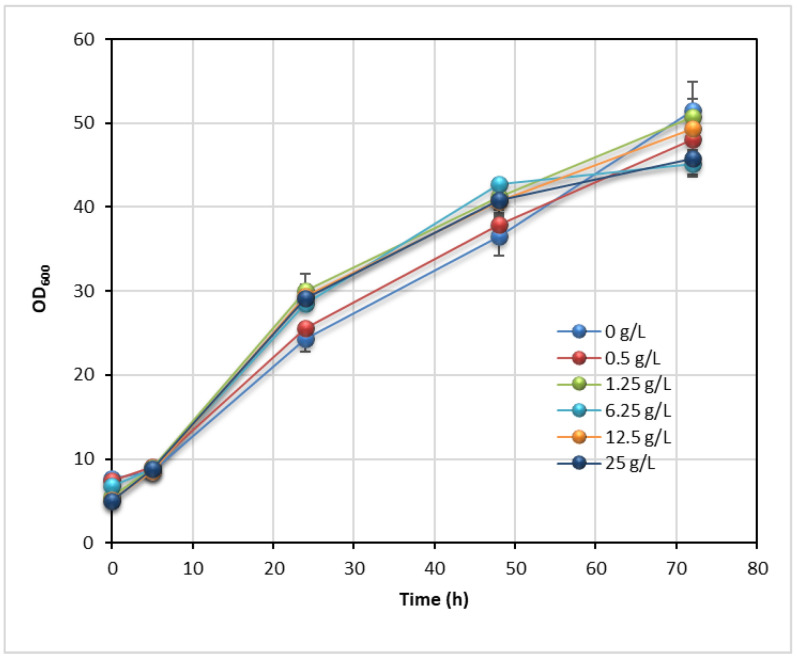
Effect of Tween 80 (0 to 25 g L^−1^) on the growth of *T. oleaginosus* on glucose-based growth medium.

The effect of Tween 80 on the growth of yeast *T. oleaginosus* and lipid accumulation was also studied. Yeast was cultivated on glucose-based media with Tween 80 over concentrations from 0 to 25 g L^−1^ (0–1.82%, *v v*^−1^). The concentrations of lipid and lipid-free cell biomass and bioprocess performance indicators are presented in Table 4. Tween 80 enhanced the lipid accumulation in *T. oleaginosus* but did not affect the cell growth (Table 4, Figure 4). During cultivation, the optical densities of yeast cultures were similar regardless of Tween 80 concentrations, indicating similar cell growth rates. A positive correlation between the final lipid concentration and Tween 80 concentration was observed. The highest lipid concentration and productivity of 6.86 g L^−1^ and 2.29 g L^−1^ d^−1^, respectively, were obtained at 25.00 g L^−1^ of Tween 80. Compared to control culture (0 g L^−1^ of Tween 80), lipid concentration and productivity increased more than 60%.

However, the lipid-free biomass concentration and productivity were insignificantly changed over the studied concentration range of surfactant (10.22–11.15 and 3.41–3.65 g L^−1^ d^−1^, respectively). A positive effect of Tween 80 on lipid synthesis has been reported for oleaginous microorganisms such as thraustochytrid *Thraustochytrium aureum* ATCC 34304 [43] and yeast *Rhodotorula glutinis* [46]. Enhancement of the rate of product synthesis in the presence of Tween 80 can be attributed to increased permeability of cell membrane [43]. Needless, some microorganisms can use Tween 80 as a carbon source for cell growth [50]. The increase of lipid content and yield suggests that *T. oleaginosus* also use Tween 80 as a carbon source for product synthesis in addition to glucose. Oleic acid, the constituent of Tween 80, can be released from surfactant by yeast esterases, transported into the cell and used as a carbon source for growth and de novo and ex novo lipid synthesis [51].

### 3.5. Fed-Batch Cultivation at High Enzyme Loading in the Presence of Tween 80

**Figure 5:**
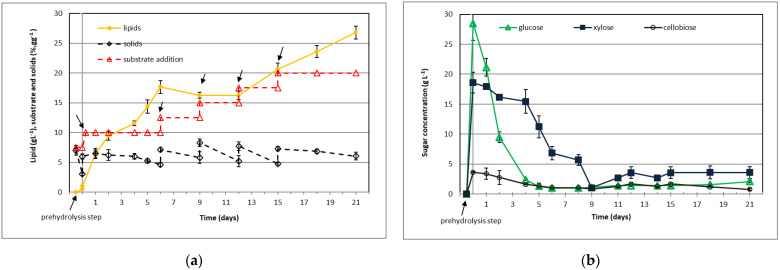
Fed-batch SSF at high enzyme loading and high C:N ratio in growth medium: (**a**) lipid and solid residue profiles and substrate loading; (**b**) fermentable sugars profiles. The arrows indicate times of substrate additions (5 × 2.5%, g g^−1^). Enzyme reaction and cultivation conditions: enzyme loading 30 FPU g^−1^ glucan, initial substrate loading 7.5% (g g^−1^), C:N ratio 207.4 mol mol^−1^, concentration of Tween 80 was 6.25 g L^−1^. Cultivation was performed in duplicate, and the values represent the average value of two parallel samples. The standard deviations between the parallel samples were below ±7% of the average values.

**Figure 6:**
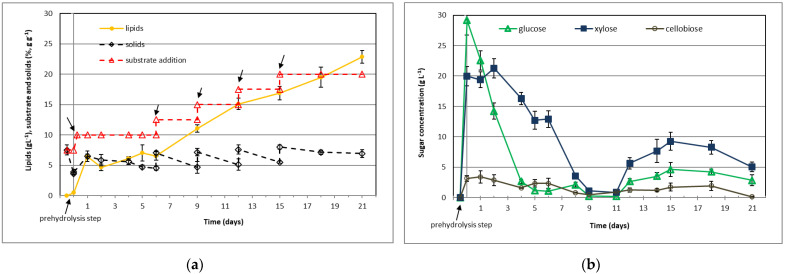
Fed-batch SSF at high enzyme loading and low C:N ratio in growth medium: (**a**) lipid and solid residue profiles and substrate loading; (**b**) fermentable sugars profiles. The arrows indicate times of substrate additions (5 × 2.5%, g g^−1^). Enzyme reaction and cultivation conditions: enzyme loading 30 FPUg^−1^ glucan, initial substrate loading 7.5% (g g^−1^), C:N ratio 38.7 mol mol^−1^, concentration of Tween 80 6.25 g L^−1^. Cultivation was performed in duplicate, and the values represent the average value of two parallel samples. The standard deviations between the parallel samples were below ±7% of the average values.

Previously, we studied the effect of substrate loading, surfactant addition, process configuration (batch and fed-batch), and substrate feed on lipid yield. Fed-batch SSF cultivation strategy with lower substrate feed (2.5% g g^−1^) doubled lipid yield (FB_2) compared to the batch SSF cultivation at the same substrate loading (B_6). Low monosaccharide concentrations in cultivation broth limited cell growth and lipid synthesis due to insufficient carbon supply. To improve lipid productivity and cell growth, we performed fed-batch SSF using a high enzyme loading of 30 FPU g^−1^ glucan. Strategies that positively affected product yield have been implemented in the next two cultivations. Fed-batch SSFs were performed under nitrogen (C:N = 207.4 mol mol^−1^; Figure 5; FB_3 cultivation) and carbon (C:N = 38.7 mol mol^−1^; Figure 6; FB_4 cultivation) limited conditions that stimulate lipid synthesis and cell proliferation, respectively [17]. After the prehydrolysis step (at 40 °C for 12 h), hydrolysates were inoculated with yeast and cultures were periodically fed with 2.5% (g g^−1^) substrate (Figure 5 and Figure 6). After five substrate additions, cumulative substrate loading was 20% (g g^−1^).

Similar profiles of solid residue concentration for both cultivations suggest a comparable hydrolysis substrate rate and growth kinetics. High glucose and xylose concentration at the beginning of the cultivations enabled fast cell growth and product accumulation. Time points of substrate additions depended on available glucose and xylose in the culture broth. During the first phase (0–4 days), glucose concentration rapidly decreased, while xylose was consumed at a lower rate, concurrently with preferred glucose. Sugar consumption correlates with rapid lipid accumulation in culture broth under an excess carbon source (Figure 5; cultivation FB_3). The highest lipid productivity of 2.71 g L^−1^ d^−1^ and lipid yield coefficient of 176 mg g^−1^ was observed on the 6th day of cultivation (Table 5; cultivation FB_3). The nitrogen excess in FB_4 decreased lipid synthesis and stimulated cell material synthesis and mitosis [8,17]. On the 9th day, the concentration of simple sugars dropped below 2 g L^−1^ in the culture broth, decreasing the rate of metabolic reactions (Figure 5 and Figure 6). In the second phase of FB_3 cultivation, low total sugar concentration (<4 g L^−1^) limited the lipid synthesis (lipid synthesis rate was 1.28 g L^−1^ d^−1^), resulting in lower lipid productivity and lipid yield coefficient (Table 5). Higher sugar concentration in FB_4 cultivation during the second growth phase supported lipid synthesis but at a lower rate (1.09 g L^−1^ d^−1^) than under nitrogen-limited conditions (FB_3). At the end of cultivation FB_4, lipid concentration was 17% lower than that under nitrogen-limited conditions due to nitrogen excess, which supported the cell growth. Consistent with the literature, a lower C:N ratio in cell culture supported cell growth, while a higher C:N ratio stimulated lipid accumulation [8,17].

Compared to batch, the fed-batch cultivation strategy of *T. oleaginosus* remarkably improved lipid yield (FB_1 and FB_2, Table 3). However, the lipid productivities were relatively low due to the limitation of growth and lipid synthesis by low sugar concentration. To improve the rate of carbohydrate hydrolysis, we applied higher enzyme loading (30 FPU g^−1^ glucan) and supplemented cultivation broth with Tween 80 in the following cultivations (FB_3 and FB_4; Figure 5 and Figure 6; Table 5). Higher sugar concentrations during the first phase of cultivation supported faster growth and lipid accumulation under nitrogen-limited conditions (FB_3), improving productivity (2.71 g L^−1^ d^−1^) and lipid concentration (17.65 g L^−1^). The sudden arrest of lipid synthesis observed on the 7th day of cultivation was probably the result of double limitation by carbon source, which dropped below 10 g L^−1^, and dissolved oxygen due to insufficient gas to liquid oxygen transfer in shake flask cultures. Under these conditions, an increase in cell death rate was expected, leading to increased cell-population heterogeneity. In the last phase of cultivation of FB_3, lipid accumulation continued at a slower rate than FB_2, resulting in lower productivity (1.28 versus 2.06 g L^−1^ d^−1^) and lipid concentration (26.8 versus 27.18 g L^−1^). Furthermore, increased enzyme loading can also decrease microorganism growth and product accumulation. A negative effect of higher loading of Celluclast 1.5 L on cell growth was observed during the cultivation of yeasts *Kluyveromyces marxianus* and *Pseudozyma* spp. [52,53]. Purification of cellulase preparation by dialysis before use improved the cell growth and product synthesis, suggesting that low-molecular-weight compounds added as preservatives and stabilizers to Celluclast 1.5 L could inhibit cell growth [52].

### 3.6. Separate Hydrolysis and Fermentation (SHF) with Cellulase Recycle

The effect of enzyme recycling on lipid production efficiency of the batch SHF process was investigated by performing successive rounds of lignocellulosic biomass hydrolysis with recycled cellulases adsorbed on unhydrolyzed solids. Substrate and enzyme loading for the first cycle was chosen based on preliminary experiments [17]. At 15% (g g^−1^) of substrate loading, more than 92% glucan and 61% of xylan were hydrolyzed to monosaccharides at a reasonably lower enzyme load of 15 FPU g^−1^. Unhydrolyzed lignocellulosic biomass from the first hydrolysis cycle with adsorbed cellulases was added to the next cycle of hydrolysis. Enzyme loading at subsequent steps (2, 3 and 4 cycles) was 90% (13.5 FPU g^−1^ glucan), 80% (12.0 FPU g^−1^) and 70% (10.5 FPU g^−1^ glucan) of enzyme loading used in the first cycle. The glucose and xylose concentrations obtained in the following steps were consistently high and comparable to the concentrations obtained after the first cycle (Table 6), suggesting that recycled cellulase and xylanase could compensate for reduced enzyme loading in cycles 2 to 4. Thus, 30% savings of cellulases can be obtained by recycling unhydrolyzed solids after enzyme hydrolysis (Table 6; cultivation B_9). After the first cycle, the values of lipid concentration and process efficiency parameters (Pr_L_ and Y_L/S_) were similar to those obtained for batch SHF at 15% (g g^−1^) substrate loading (cultivation B_5). In the following cycles, lipid concentrations were between 10.33 and 12.87 g L^−1^ regardless of enzyme loading, while lipid productivity and lipid yield remained almost constant (cultivations B-7, B-8 and B-9).

The principal advantage of SHF process is performing enzyme hydrolysis and microorganism growth at optimal conditions (temperature and pH) [17,24]. However, the productivity of the batch SHF processes of lipid production is generally lower than SSF process due to inhibition of microorganism growth by high substrate concentration at the beginning of the cultivation and longer process times due to the separate enzyme hydrolysis step [17].

### 3.7. Mass Balances of Lipid Production

**Figure 7:**
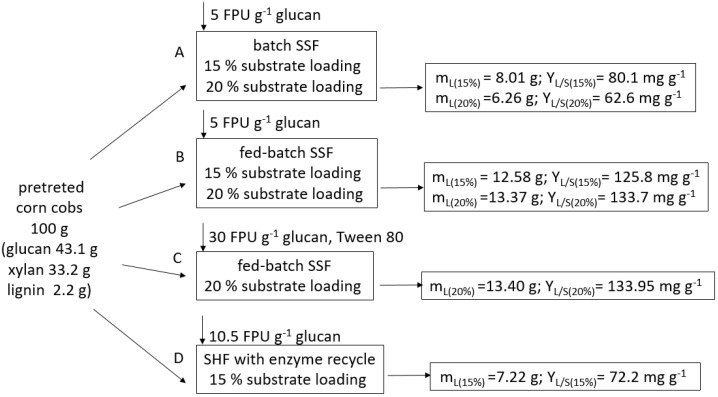
Mass balance of lipid production by *T. oleaginosus* on hydrolysate of pretreated corn cobs using different cultivation strategies. (m_L_, lipid weight; Y_L/S_, lipid yield on pretreated lignocellulosic biomass).

Mass balances of the four processes for lipid production from pretreated corn cobs using four paths are summarized in Figure 7. Alkaline pretreated corn cobs contained 43.1 g of glucan, 33.2 xylan and 2.2 g of total lignin (acid-soluble and acid-insoluble lignin). From 100 g of pretreated corn cobs, 8.01 and 6.2 g of lipids were produced by batch SHF at 15 and 20% (g g^−1^) substrate loading with lipid yields of 80.1 and 62.6 g g^−1^, respectively (path A). Increasing substrate loading from 15 to 20% (g g^−1^) decreased lipid yield and slightly increased the product weight. When the fed-batch strategy was applied (path B), lipid yield increased to 125.8 and 133.7 mg g^−1^ using cumulative substrate loading of 15 and 20% (g g^−1^), respectively. Lipid weight produced on 100 g of pretreated corn cobs was doubled compared to batch SSF (path A). When high cellulase loading was applied (path C), lipid yield and lipid weight in fed-batch SSF was not improved (13.4 g and 133.95 mg g^−1^, respectively). Batch SHF strategy of lipid production (path D) with enzyme recycle produced only 7.2 g of lipids with a yield of 72.2 mg g^−1^ of pretreated corn cobs. Despite the enzyme savings, the cellulase loading in SHF (path D) was significantly higher than that in the fed-batch SSF process (path B). Obtain results suggest that fed-batch SSF lipid production (path B and C) enables higher lipid yield compared to SHF with enzyme recycle (path D) and batch SSF (path A). Furthermore, enzyme loading was six times lower in path B compared to path C. It would be economically attractive to produce the lipid using fed-batch SSF.

## 4. Conclusions

Process strategy of microbial lipid production from lignocellulosic biomass affects the lipid yield and productivity. Fed-batch SSF is the most efficient and economically feasible among the studied approaches. Further improvement of cultivation condition, substrate feeding strategy, mass transfer in culture broth and cellulase hydrolysis is crucial for establishing efficient and sustainable lignocellulosic biorefinery. Recycling of adsorbed cellulase could replace part of the fresh enzyme in SHF (up to 30%) and reduce production costs without significantly affecting lipid yield and productivity.

## Figures and Tables

**Table 1:** Composition of untreated and alkali-pretreated corn cobs.

	Glucan (%, g g^−1^)	Xylan (%, g g^−1^)	Lignin Insoluble in Acid (%, g g^−1^)	Lignin Soluble in Acid (%, g g^−1^)
untreated	38.6 ± 4.44	36.8 ± 0.65	13.9 ± 0.37	0.4 ± 0.30
alkali-pretreated	43.1 ± 5.08	33.2 ± 3.66	2.1 ± 1.08	0.1 ± 0.08

**Table 2:** Effect of substrate loading (5.0–20.0%**,** g g^−1^) on lipid production in batch SSF at low enzyme loading (5 FPU g^−1^ glucan).

Cultivation	Substrate (%, g g^−1^)	Time (d)	w_L_ (%, g g^−1^)	L (g L^−1^)	Y_L/S_ (mg g^−1^)	Pr_L_ (g L^−1^ d^−1^)	η_L_ (%)
B_1	5.0	3	9.10 ± 0.76	2.99 ± 0.61	59.72	0.99	21.23
B_2	7.5	6	13.36 ± 0.60	5.56 ± 0.61	74.07	0.93	26.33
B_3	10.0	10	20.98 ± 0.66	7.47 ± 0.58	74.7	0.75	26.55
B_4	12.5	10	14.37 ± 0.24	12.00 ± 0.48	92.1	0.88	32.72
B_5	15.0	10	14.50 ± 0.43	12.02 ± 0.87	80.1	1.20	28.47
B_6	20.0	10	9.27 ± 0.17	12.52 ± 0.52	62.6	1.25	22.25

w_L_, lipid content in solid residue; L, lipid concentration; Y_L/S_, lipid yield on pretreated lignocellulosic biomass; Pr_L_, maximal lipid productivity; η_L_, lipid recovery on pretreated lignocellulosic biomass (calculated according to Ivancic Santek et al. [17]).

**Table 3:** Fed-batch SSF at low enzyme loading. Initial substrate loading was 5% (g g^−1^); enzyme loading was 5 FPU g^−1^ glucan.

	Time (d)	Substrate		Solid Residue (g L^−1^)	w_L_ (%, g g^−1^)	L (g L^−1^)	Y_L/S_ (mg g^−1^)	Pr (g L^−1^ d^−1^)	η_L_ (%)
		No. Batch Additions	Cumulative (%, g g^−1^)						
FB_1	0	2 × 5	5	4.94 ± 0.42	-	-	-	-	-
	7	% (g g^−1^)	10	4.98 ± 0.77	20.25 ± 1.43	10.08 ± 2.20	100.8	1.44	35.83
	8		15	8.24 ± 0.70	14.15 ± 1.00	11.66 ± 1.76	77.7	1.46	27.63
	10		15	8.12 ± 2.15	23.23 ± 2.63	18.87 ± 2.46	125.8	1.89	44.71
FB_2	0	6 × 2.5	5	4.96 ± 0.28	-	-	-	-	-
	6	% (g g^−1^)	10	5.76 ± 0.41	14.83 ± 0.80	8.54 ± 0.12	85.40	1.42	30.36
	10		15	7.99 ± 0.68	24.47 ± 1.73	19.54 ± 0.34	130.3	1.95	46.30
	13		20	9.84 ± 0.56	27.18 ± 0.77	26.74 ± 2.31	133.7	2.06	47.52

w_L_, lipid content in solid residue; L, lipid concentration; Y_L/S_, lipid yield on pretreated lignocellulosic biomass; Pr, lipid productivity; η_L_, lipid recovery on pretreated lignocellulosic biomass (calculated according to Ivancic Santek et al. [17]).

**Table 4:** Effect of Tween 80 on the growth of yeast *T. oleaginosus* and lipid production on glucose-based growth medium.

Tween 80 (g L^−1^)	X* (g L^−1^)	L (g L^−1^)	Y_L/S_ (g g^−1^)	Y_X/S_ (g g^−1^)	η_L_ (%)	Pr_L_ (g L^−1^ d^−1^)	Pr_X_ (g L^−1^ d^−1^)
0.00	10.22 ± 0.78	4.36 ± 0.21	0.109	0.256	34.2	1.45	3.41
0.50	10.48 ±0.54	4.70 ± 0.28	0.117	0.260	36.5	1.57	3.49
1.25	10.42 ± 0.98	4.50 ± 0.16	0.110	0.255	34.5	1.50	3.47
2.50	10.53 ± 0.62	5.24 ± 0.27	0.126	0.252	39.3	1.75	3.51
6.25	10.95 ± 0.53	5.07 ± 0.23	0.122	0.264	38.2	1.69	3.65
12.50	11.15 ± 0.31	5.43 ± 0.41	0.129	0.266	40.4	1.81	3.72
25.00	10.70 ± 0.61	6.86 ± 0.31	0.161	0.251	50.4	2.29	3.57

X*, concentration of lipid-free yeast biomass (X* = X_T_⋅(100 − w_L_)/100, X_T_ total cell concentration, w_L_, lipid content in cell biomass); L, lipid concentration; Y_L/S_, lipid yield on pretreated lignocellulosic biomass; Y_X/S_, lipid-free cell biomass yield on pretreated lignocellulosic biomass; Pr_L_, lipid productivity; Pr_X_, biomass productivity; η_L_, lipid recovery on pretreated lignocellulosic biomass (calculated according to Ivancic Santek et al. [17]).

**Table 5:** The efficiency of the fed-batch SSF process at high enzyme loading (30 FPU g^−1^ glucan) in the presence of Tween 80 under nitrogen-limited (FB_3) and carbon limited conditions (FB_4). The growth medium was supplemented with 6.25 g L^−1^ Tween 80. Initial substrate loading was 7.5% (g g^−1^).

	Time (d)	Cumulative Substrate Loading (%, g g^−1^)	Solid Residue (%, g g^−1^)	L (g L^−1^)	Y_L/S_ (mg g^−1^)	Pr_L_ (g L^−1^ d^−1^)	η_L_ (%)
FB_3	6	10	4.61 ± 0.33	17.65 ± 1.06	176.5	2.71	62.81
	21	20	6.05 ± 0.63	26.8 ± 1.03	133.95	1.28	47.61
FB_4	6	10	4.52 ± 0.31	7.02 ± 0.69	70.2	1.02	25.62
	21	20	6.92 ± 0.62	22.8 ± 1.03	114.2	1.09	40.6

L, lipid concentration; Y_L/S_, lipid yield on pretreated lignocellulosic biomass; Pr_L_, lipid productivity; η_L_, lipid recovery on pretreated lignocellulosic biomass (calculated according to Ivancic Santek et al. [17]).

**Table 6:** Lipid production by batch SHF with enzyme recycle. Lignocellulose hydrolysis conditions: substrate loading 15% (g g^−1^); enzyme loading: 15 FPU g^−1^ glucan in the cycle 1; 13.5 (90%), 12.0 (80%) and 10.5 (70%) FPU g^−1^ glucan in the cycles 2–4. Unhydrolyzed lignocellulosic biomass from the previous hydrolysis cycle was added to new lignocellulosic biomass in the next hydrolysis cycle.

	Cycle (no.)	Enzyme Loading (%) *	Glucose (g L^−1^)		Xylose (g L^−1^)		X_T_ (g L^−1^)	w_L_ (%, g g^−1^)	L (g L^−1^)	Y_L/S_ (mg g^−1^)	Pr_L_ (g L^−1^ d^−1^)	η_L_ (%)
			after Enzyme Hydrolysis	after Cultivation	after Enzyme Hydrolysis	after Cultivation						
B_7	1	100	67.80 ± 4.28	4.33 ± 3.19	35.12 ± 1.51	21.89 ± 1.17	25.21 ± 1.38	48.27 ± 5.45	12.17	0.081	1.134	28.8
	2	90	61.67 ± 0.92	1.58 ± 0.29	37.90 ± 0.71	24.98 ± 0.05	23.65 ± 2.44	44.96 ± 0.80	10.63	0.071	0.994	25.2
	3	90	62.26 ± 0.69	1.83 ± 2.59	39.54 ± 0.77	28.35 ± 5.16	28.20 ± 4.26	45.07 ± 0.88	12.71	0.085	1.19	30.1
	4	90	62.91 ± 0.25	4.27 ± 1.22	39.89 ± 0.41	29.15 ± 0.16	24.58 ± 0.98	47.69 ± 2.48	11.72	0.078	1.092	27.8
B_8	1	100	68.63 ± 4.73	0.37 ± 0.52	36.37 ± 1.92	17.63 ± 4.83	26.01 ± 3.61	49.48 ± 1.65	12.87	0.086	1.204	30.5
	2	80	62.07 ± 0.48	3.94 ± 0.79	37.76 ± 0.10	27.80 ± 0.34	23.41 ± 0.98	44.12 ± 2.10	10.33	0.069	0.966	24.5
	3	80	61.96 ± 0.84	0.83 ± 1.17	39.29 ± 0.06	24.61 ± 1.20	26.95 ± 0.25	45.07 ± 0.88	12.15	0.081	1.134	28.8
	4	80	61.81 ± 0.14	3.97 ± 1.70	39.61 ± 0.70	26.42 ± 3.39	25.23 ± 3.16	47.16 ± 6.69	11.90	0.079	1.106	28.2
B_9	1	100	65.99 ± 3.90	0.00 ± 0.00	34.56 ± 1.95	18.40 ± 0.60	23.88 ± 3.34	48.24 ± 1.60	11.52	0.077	1.078	27.3
	2	70	59.93 ± 1.44	4.41 ± 1.66	35.72 ± 1.17	23.35 ± 0.31	24.48 ± 1.43	44.10 ± 1.15	10.80	0.072	1.008	25.6
	3	70	61.43 ± 2.15	1.82 ± 0.46	38.55 ± 1.26	25.96 ± 0.12	23.30 ± 1.55	46.01 ± 3.04	10.72	0.071	0.994	25.4
	4	70	61.64 ± 0.26	2.02 ± 6.65	39.29 ± 0.10	25.86 ± 1.59	23.87 ± 0.91	46.64 ± 3.21	11.13	0.074	1.036	26.4

X_T_, total cell biomass concentration (lipid-free cell biomass concentration+lipid concentration); w_L_, lipid content in cell biomass; L, lipid concentration; Y_L/S_, lipid yield on pretreated lignocellulosic biomass; Pr_L_, lipid productivity; η_L_, lipid recovery on pretreated lignocellulosic biomass (calculated according to Ivancic Santek et al. [17]).

## Data Availability

Data is contained within the article.
